# Isopsoralen Enhanced Osteogenesis by Targeting AhR/ERα

**DOI:** 10.3390/molecules23102600

**Published:** 2018-10-11

**Authors:** Luna Ge, Yazhou Cui, Kai Cheng, Jinxiang Han

**Affiliations:** 1 School of Traditional Chinese Medicine, Shandong University of Traditional Chinese Medicine, Jinan 250355, China; geluna606@163.com; 2 Shandong Medicinal Biotechnology Center, Key Laboratory for Biotech-Drugs of the Ministry of Health, Jinan 250062, China; cuiyz15@126.com; 3 Shandong Academy of Medical Sciences, Jinan 250062, China; chengkai606@163.com

**Keywords:** isopsoralen, osteogenesis, aryl hydrocarbon receptor, estrogen receptor alpha

## Abstract

Isopsoralen (IPRN), one of the main effective ingredients in *Psoralea corylifolia* Linn, has a variety of biological effects, including antiosteoporotic effects. In vivo studies show that IPRN can increase bone strength and trabecular bone microstructure in a sex hormone deficiency-induced osteoporosis model. However, the mechanism underlying this osteogenic potential has not been investigated in detail. In the present study, we investigated the molecular mechanism of IPRN-induced osteogenesis in MC3T3-E1 cells. Isopsoralen promoted osteoblast differentiation and mineralization, increased calcium nodule levels and alkaline phosphatase (ALP) activity and upregulated osteoblast markers, including ALP, runt-related transcription factor 2 (RUNX2), and collagen type I alpha 1 chain (COL1A1). Furthermore, IPRN limited the nucleocytoplasmic shuttling of aryl hydrocarbon receptor (AhR) by directly binding to AhR. The AhR target gene cytochrome P450 family 1 subfamily A member 1 (CYP1A1) was also inhibited in vitro and in vivo. This effect was inhibited by the AhR agonists indole-3-carbinol (I3C) and 3-methylcholanthrene (3MC). Moreover, IPRN also increased estrogen receptor alpha (ERα) expression in an AhR-dependent manner. Taken together, these results suggest that IPRN acts as an AhR antagonist and promotes osteoblast differentiation via the AhR/ERα axis.

## 1. Introduction

Osteoporosis is a global health problem that seriously affects the quality of patients’ lives. As aging-associated problems have increased, at least one-quarter of people older than 60 years have different levels of osteoporosis [1]. Osteoporosis is a systemic skeletal disease mainly characterized by bone loss, bone microstructure degradation, reduced bone strength, and increased fracture risk [2]. Bone metabolism includes both bone formation and resorption.

A large number of clinical and experimental studies have confirmed the positive effect of estrogen replacement therapy on the prevention and treatment of postmenopausal osteoporosis, but this treatment also increases the relative risk of cardiovascular diseases, and long-term application can cause breast cancer and endometrial cancer [3]. Therefore, finding an ideal drug to prevent and treat postmenopausal osteoporosis with few side effects is currently an active and difficult research topic.

**Figure 1:**
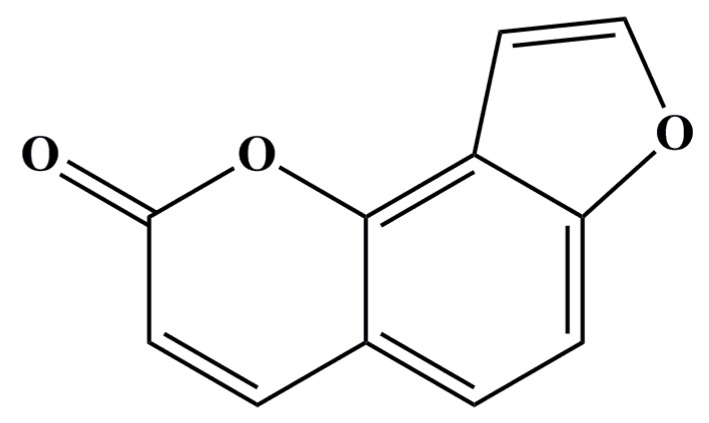
The structure of isopsoralen (IPRN).

As a kind of coumarin compound, isopsoralen (IPRN) (Figure 1) is the main active component of *Psoralea corylifolia Linn* and has antibacterial, anti-inflammatory, and antitumor activities [4,5]. A previous experiment indicated that IPRN could act against hormone deficiency osteoporosis [6]. Isopsoralen exhibits estrogen-like activities and performs estrogen-like neuroprotection against spinal cord injury-induced apoptosis by activating estrogen receptor alpha (ERα) [7]. ERα promotes osteoblasts differentiation and the lack of ERα reduces longitudinal bone growth during sexual maturation both in male and female mice [8,9]. Previous studies have also shown that IPRN inhibits the activity of cytochrome P450 family 1 subfamily A member 2 (CYP1A2), which is one of the target genes of aryl hydrocarbon receptor (AhR). The AhR acts as a negative regulator of bone mass by suppressing osteoblasts proliferation and differentiation [10,11]. Therefore, we hypothesized that IPRN promoted osteoblasts differentiation and mineralization through AhR and ERα. In this study, MC3T3-E1 cells were used as a model to determine the mechanism of IPRN acting on osteoblast differentiation. In the current study, we found that IPRN could promote osteogenic differentiation and mineralization in a dose-dependent manner by limiting the nucleocytoplasmic shuttling of AhR. Isopsoralen could directly bind to AhR and inhibit the expression of ERα.

## 2. Results

### 2.1. IPRN Promoted the Osteogenic Differentiation and Mineralization of MC3T3-E1 Cells

**Figure 2:**
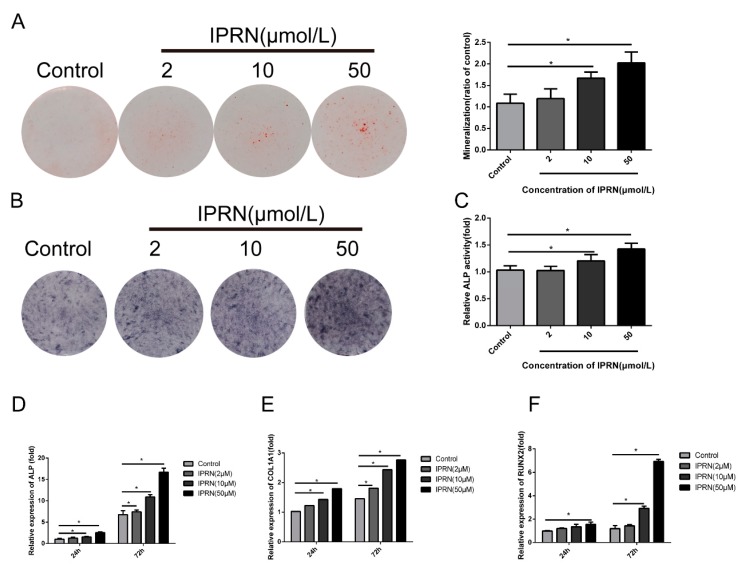
Effects of IPRN on osteoblastic differentiation of MC3T3-E1 cells. Cells were cultured in osteogenic medium with the indicated concentration of IPRN (2–50 μM) for 14 days (**A**), 9 days (**B**,**C**) or 24 and 72 h (**D**–**F**). (**A**) Alizarin red staining was performed to assess mineralization, and the cells were then solubilized with 10% cetylpyridinium chloride and quantified at 562 nm. Differentiation was assessed by alkaline phosphatase (ALP) staining (**B**), ALP activity (**C**) and the mRNA expression of the osteogenic markers *ALP* (**D**), collagen type I alpha 1 chain (*COL1A1*) (**E**), and runt-related transcription factor 2 (*RUNX2*) (**F**). Data are presented as the mean ± standard deviation (SD) (*n* = 3). Experiments in this figure were repeated three times, and similar results were obtained. * *p* < 0.05 vs. control.

Previous studies showed that IPRN could promote osteogenesis in vivo. To investigate the molecular mechanism, we evaluated the osteogenic differentiation and mineralization induced by IPRN in MC3T3-E1 subclone 14 cells. Cells were cultured in osteogenic medium with different concentrations of IPRN (0, 2, 10, or 50 μM). The Alizarin red staining results indicated that IPRN increased the production of calcium nodules in a concentration-dependent manner after 14 days of treatment (Figure 2A). The formation of calcium nodules is one of the indicators of osteoblastic maturation. The effect of IPRN on osteogenic differentiation was evaluated via alkaline phosphatase (ALP) activity, ALP staining, and osteoblast differentiation markers. As shown in Figure 2B, IPRN promoted ALP activity in a dose-dependent manner, as indicated by ALP staining, and the effect was also confirmed by an ALP activity assay (Figure 2C). Alkaline phosphatase (ALP) helps to produce a mineralized matrix in osteoblasts. Furthermore, the expression of osteoblastic genes was assessed by RT-qPCR. Compared with the control group, the groups treated with IPRN had significantly higher expression of ALP, runt-related transcription factor 2 (RUNX2), and collagen type I alpha 1 chain (COL1A1) (Figure 2D–G).

### 2.2. IPRN Limited the Nucleocytoplasmic Shuttling of AhR

**Figure 3:**
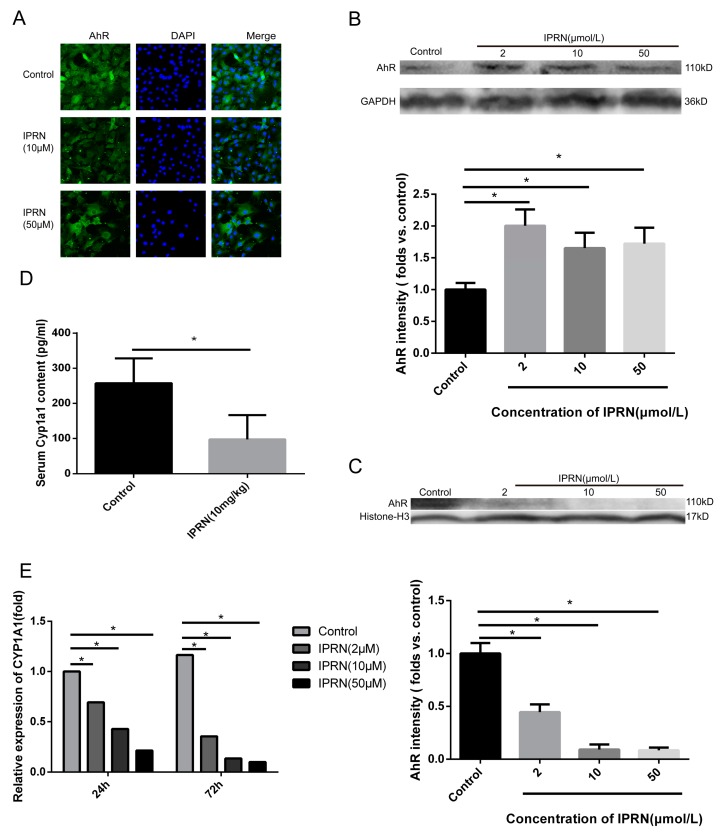
IPRN acted as an AhR antagonist in MC3T3-E1 cells. Immunofluorescence staining (**A**) for AhR was performed after treatment with IPRN for 24 h. The protein levels of AhR in the cytoplasm (**B**) and nucleus (**C**) were assessed by Western blotting. CYP1A1 mRNA expression (**D**) was determined by RT-qPCR (*n* = 3). (**E**) CYP1A1 levels were also measured in vivo in the serum of mice after IPRN (10 mg/kg) treatment (*n* = 6). Data are presented as the mean ± SD. * *p* < 0.05 vs. control.

Previous studies have shown that IPRN can inhibit the activity of CYP1A2, which is one of the target genes of AhR [12,13]. The AhR acts as a transcription factor and plays an important role in a series of physiological processes, including bone development [14]. Therefore, we hypothesized that IPRN may increase the expression of osteogenic proteins by targeting AhR. First, immunofluorescence staining was performed to determine the expression change in AhR after IPRN treatment. As shown in Figure 3A, compared to the control, IPRN decreased the nucleocytoplasmic shuttling of AhR. Then, the protein levels of AhR in the cytoplasm and nucleus were assessed. Consistent with the immunofluorescence staining results, AhR was retained in the cytoplasm, and AhR in the nucleus was decreased after IPRN treatment (Figure 3B,C). The mRNA expression and activity of CYP1A1, which is the main target gene of AhR, was also detected in vitro and in vivo. Isopsoralen inhibited the mRNA expression of CYP1A1 in a dose-dependent manner after treatment for 24 h and 72 h (Figure 3D). To confirm the effect in vivo, C57BL/c mice were injected with IPRN at a dose of 10 mg/kg to evaluate CYP1A1 activity in serum. Compared to the control group, IPRN significantly downregulated CYP1A1 activity (Figure 3E).

### 2.3. AhR Could Directly Bind to IPRN

**Figure 4:**
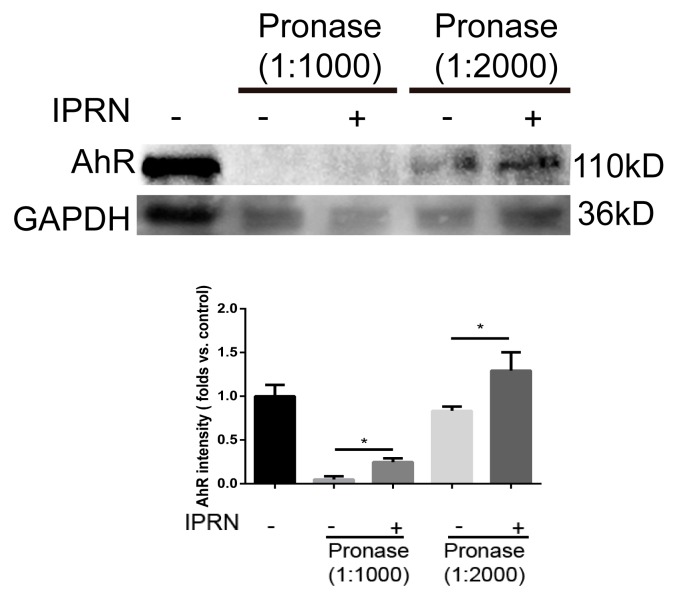
IPRN directly binds to AhR. For the DARTS assay, MC3T3-E1 cell lysates (5 mg/mL) were incubated with IPRN (10 μM) or an equal volume of DMSO for 1 h at room temperature, followed by digestion with pronase to protein ratios of 1:1000 or 1:2000 for 30 min. The samples were analyzed using Western blotting. Data are presented as the mean ± SD (*n* = 3). * *p* < 0.05.

Because IPRN suppressed the nuclear translocation of AhR and the transcription of CYP1A1, we hypothesized that IPRN may improve osteogenic differentiation via direct binding to AhR. To determine whether IPRN could directly bind to AhR, drug affinity responsive target stability (DARTS) was performed. Drug affinity responsive target stability is a useful method for the initial identification of the protein targets of small molecules [15,16]. Drug affinity responsive target stability analysis revealed that IPRN could directly bind to AhR and inhibit its proteolysis (Figure 4).

### 2.4. AhR Agonists Inhibited IPRN-Induced Osteogenic Activity

**Figure 5:**
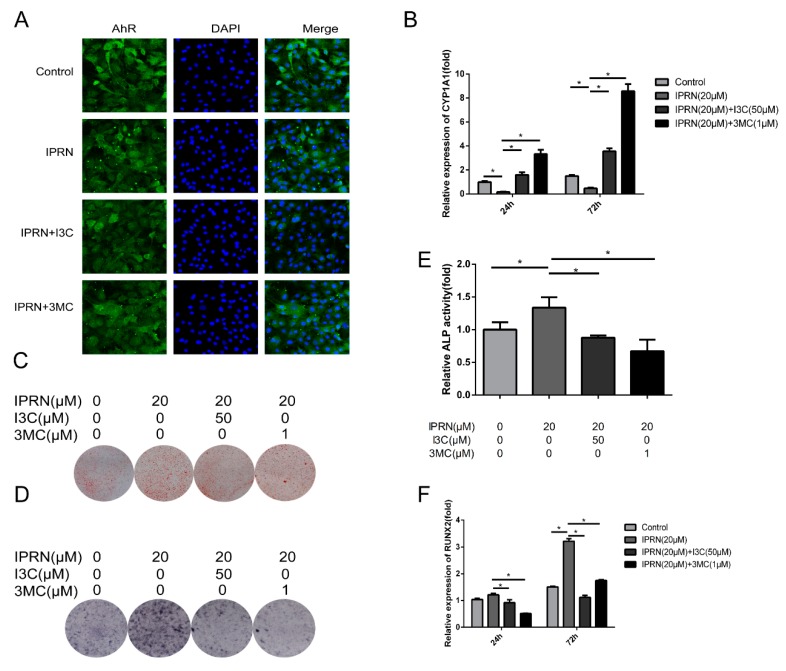
Effect of AhR agonists on IPRN-induced osteogenesis. The cells were cultured with I3C (50 μM) or 3MC (1 μM) in the presence of IPRN (20 μM). (**A**) Immunofluorescence analysis of AhR was performed at 24 h. CYP1A1 (**B**) and RUNX2 (F) mRNA expression was assessed at 24 h and 72 h, respectively. Alizarin red (**C**) and ALP (**D**) staining was performed and ALP activity (**E**) was assessed on days 14 and 9, respectively. Data are presented as the mean ± SD (*n* = 3). * *p* < 0.05.

To further confirm the role of AhR in IPRN-induced stimulation of osteoblast differentiation, AhR agonists were used for cotreatment with IPRN. The IPRN-induced inhibition of nucleocytoplasmic shuttling of AhR was suppressed by I3C and 3MC (Figure 5A). The mRNA expression of CYP1A1 was also increased after the cotreatment of I3C and 3MC (Figure 5B). Furthermore, the mineralization and osteogenic differentiation induced by IPRN were all suppressed after treatment with AhR agonists (Figure 5C–F).

### 2.5. IPRN Promoted the Expression of ERα in An AhR-Dependent Manner

**Figure 6:**
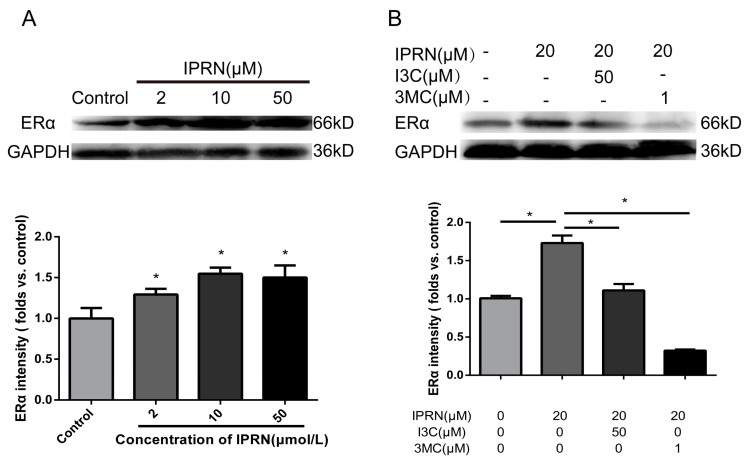
Effects of IPRN and AhR agonists on ERα expression. The cells were cultured with IPRN (**A**) or AhR agonists (**B**) in osteogenic medium for 5 days. Data are presented as the mean ± SD (*n* = 3). * *p* < 0.05.

Apart from acting as a transcription factor, AhR also acts as an E3 ubiquitin ligase to modulate levels of steroid receptor proteins, such as ERα [17,18]. ERα acts as a nuclear receptor that can be activated by the sex hormone estrogen. During bone development, the knockout of AhR induces decreased bone length and size [19]. Therefore, whether IPRN stimulated the expression of ERα was determined. As shown in Figure 6A, IPRN increased the protein level of ERα in a dose-dependent manner. The effect was inhibited by the AhR agonists I3C and 3MC (Figure 6B).

## 3. Discussion

The AhR is a ligand-activated transcription factor that belongs to the bHLH-PAS family [20]. Upon binding to agonists, AhR undergoes a structural change and translocates to the nucleus. Then, AhR dimerizes with aryl hydrocarbon receptor nuclear translocator (ARNT) and binds to the xenobiotic responsive element (XRE) sequence, inducing the expression of its target genes, such as CYP1A1 and CYP1A2 [21].

Previous studies have shown that IPRN is a potent time-dependent inhibitor of CYP1A2 in vitro and in vivo. Therefore, we speculated that IPRN acts as an AhR antagonist. Immunochemical staining was performed in MC3T3-E1 cells after treatment with IPRN for 24 h. Compared to that in the control group, the nuclear-cytoplasmic trafficking of AhR in osteoblasts was restrained by IPRN (Figure 3A). After IPRN treatment, AhR levels significantly decreased in the nucleus but increased in the cytoplasm (Figure 3B,C). To further confirm this result, the expression of CYP1A1, which is the main target gene of AhR, was determined in vitro and in vivo. CYP1A1 mRNA was significantly decreased in MC3T3-E1 cells after treatment with IPRN for 24 h and 72 h (Figure 3D). The same results were also observed in C57BL/c mice (Figure 3E). These results suggest that IPRN acts as an AhR antagonist. To confirm whether IPRN could directly bind to AhR, a DARTS assay was performed. As shown in Figure 4, IPRN could directly bind to AhR and inhibit its proteolysis. To our knowledge, this study is the first to show that IPRN can directly bind to AhR and inhibit its nuclear translocation.

Previous studies on AhR have largely focused on mediating xenobiotic toxicities induced by toxic environmental contaminants, such as tetrachlorodibenzo-p-dioxin (TCDD). However, recent studies have shown that AhR participates in a wide variety of important physiological and pathological processes. In skeletal development and homeostasis, AhR exerts a negative regulatory effect [10,22]. The activation of AhR induced by TCDD prevents the proliferation and osteogenic differentiation of MC3T3-E1 cells [11]. In the current study, the stimulation of osteogenic differentiation induced by IPRN could be prevented by I3C and 3MC, which act as AhR agonists.

Apart from acting as a transcription factor, AhR also acts as a ligand-dependent E3 ubiquitin ligase that affects many signaling pathways. Activation of AhR induces proteasome-dependent ERα degradation in human breast cancer cells [23]. The ERα plays an important role in the process of skeletal development. Estrogens can regulate the life span of osteoblasts and osteocytes through ERα. Female mice lacking ERα display compromised bone mass and strength [24]. In recent years, selective estrogen receptor modulators (SERMs) have been applied in the treatment of postmenopausal osteoporosis [25,26]. In our study, IPRN significantly increased the expression of ERα, and this effect was inhibited by AhR agonists. These results showed that IPRN promotes the differentiation and mineralization of MC3T3-E1 cells through the AhR/ERα pathway.

## 4. Materials and Methods

### 4.1. Animals and Chemicals

Eight-week-old C57BL/c mice were purchased from Jinan Pengyue Experimental Animal Breeding Co. Ltd. (Jinan, China) (license number: SCXK (Lu) 20140007). All experimental protocols in the current study were approved by the Institutional Animal Care and Use Committee of Shandong Academy of Medical Sciences. IPRN was obtained from Chengdu Herbpurify Co., Ltd. (Chengdu, China). I3C was obtained from Medchem Express (Princeton, NJ, USA). All other chemicals were obtained from Sigma-Aldrich Company (St. Louis, MO, USA). Antibodies against RUNX2 and AhR were obtained from Cell Signaling Technology (Danvers, MA, USA). All other antibodies were purchased from Proteintech (Wuhan, China).

### 4.2. MC3T3-E1 Cell Culture

The MC3T3-E1 subclone 14 cell line was obtained from the Cell Bank of Type Culture Collection of the Chinese Academy of Sciences (Shanghai, China) and cultured in α-MEM (HyClone, Logan, UT, USA) supplemented with 10% fetal bovine serum (Gibco, Waltham, MA, USA) and 1% penicillin-streptomycin in 5% CO_2_ at 37 °C. The osteogenic medium was supplemented with 10 mM β-glycerophosphate and 50 μg/mL ascorbic acid-2-phosphate.

### 4.3. Alizarin Red and ALP Staining

MC3T3-E1 cells were cultured in 24-well plates and incubated with osteogenic medium supplemented with various concentrations of IPRN, I3C and 3MC. The cells were fixed with 4% paraformaldehyde for 30 min at room temperature. Then, the Alizarin red and ALP stains were prepared with 0.5% Alizarin red S solution (pH 4.2) and a BCIP/NBT alkaline phosphatase color development kit (Beyotime, Shanghai, China), respectively. Images of the stained cells were captured with a digital camera.

### 4.4. ALP Activity Measurement

The cells treated in osteogenic medium for 9 days in 24-well plates were washed twice with PBS and lysed in 0.1% Triton X-100 buffer on ice for 2 h. Then, the lysates were centrifuged at 12,000 rpm for 15 min at 4 °C. The total protein was measured with a BCA protein assay kit (Beyotime, Shanghai, China). P-nitrophenyl phosphate (Sigma-Aldrich, St. Louis, MO, USA) was used as the substrate to evaluate the ALP activity. The absorbance was measured at 405 nm and normalized to the total protein.

### 4.5. RT-qPCR

Osteoblasts were seeded in 6-well plates and treated with angelicin (0, 0.1, 1 or 10 μM). Trizol reagent (Invitrogen, Albuquerque, NM, USA) was added to the cells seeded in 6-well plates to extract the total RNA according to the manual. Then, the RNA samples were reverse transcribed into cDNAs using the ReverTra Ace^®^ qPCR RT Kit (Toyobo, Shanghai, China) at 37 °C for 15 min and 98 °C for 5 min. Real-time quantitative polymerase chain reaction (qRT-PCR) was performed on a LightCycler^®^ 480II real-time PCR system (Roche, Mannheim, Germany) using relative quantitation gene expression assays (Nova, Lianyungang, China). The thermocycling conditions were as follows: 94 °C for 3 min followed by 40 cycles of 94 °C for 15 s and 64 °C for 1 min. All reactions were carried out in triplicate, and the mRNA expression level was calculated using the 2^−ΔΔCq^ method with normalization to GAPDH. The primer sequences are listed in Table 1.

### 4.6. Western Blotting

Cells seeded in 75 cm^2^ culture bottles were lysed with cell lysis buffer for Western blotting or for the nuclear and cytoplasmic protein extraction kit according to the manufacturer’s instructions. A BCA assay kit was used to measure the protein concentrations. Proteins (40 µg) were separated by 10% SDS-PAGE and transferred to 0.45 µm PVDF membranes. Membranes were blocked with 5% skim milk for 1 h at room temperature and incubated with the appropriate primary antibodies (Runx2, AhR, ERα, histone H3, and GAPDH) at 4 °C overnight. The membranes were subsequently washed three times with TBST and incubated with HRP-labeled goat anti-rabbit IgG for 1 h at room temperature. Blots were washed again with TBST and visualized using an enhanced ECL substrate kit. The densities of the product bands were quantified using Image J software and standardized against GAPDH or histone H3.

### 4.7. Immunochemical Staining

Immunochemical staining was performed as described previously [27]. Cells seeded in 48-well plates were fixed with 4% paraformaldehyde for 15 min and washed three times with PBS. Then, the cells were permeabilized with 0.3% Triton X-100 for 30 min followed by blocking with Immunol Staining Blocking Buffer (Beyotime, Shanghai, China) for 1 h at room temperature. The cells were incubated with anti-AhR antibody (1:100, Proteintech) overnight at 4 °C and rewarmed at 37 °C for 1 h the next day. The cells were incubated with Alexa-488-conjugated secondary antibody (1:500, Proteintech) for 50 min at 37 °C, stained with DAPI (Beyotime, Shanghai, China), washed three times, and viewed by a laser scanning confocal microscope (Olympus, Tokyo, Janpan).

### 4.8. DARTS Analysis

Cells seeded in 75 cm^2^ culture bottles were lysed with M-PER lysis buffer (Thermo Scientific Pierce, Waltham, MA, USA) supplemented with phosphatase and protease inhibitors. The cell lysates were centrifuged at 16,000 *g* for 20 min at 4 °C. A total of 600 μL of supernatant was transferred into a new 1.5 mL tube, and 66.7 µL of 10 × TNC buffer was added. The lysates were split into two samples after the protein concentration measurement. The two samples were incubated with DMSO or IPRN (100 μM) at room temperature for 1 h. Two aliquots from both protein samples were incubated with 1:1000 and 1:2000 pronase solution at room temperature for 30 min. Sodium dodecyl sulfate (SDS) loading buffer was added to stop the proteolysis. Finally, all the samples were analyzed by immunoblotting.

### 4.9. Statistical Analysis

All data are expressed as the mean ± standard deviation (SD). One-way ANOVA statistical analysis was conducted followed by Tukey’s test for multiple comparisons if necessary. In all cases, *p* < 0.05 was considered significant.

## 5. Conclusions

The present study was conducted to investigate the osteogenesis induced by IPRN. The results showed that IPRN could directly bind to AhR and act as an AhR antagonist. Moreover, IPRN increased the expression of ERα, and this effect depended on AhR. Our studies shed light on the osteogenic effect of IPRN that occurs through the AhR/ERα pathway. This study provides a novel mechanism of IPRN-induced osteogenesis and provides a theoretical basis for clinical application.

## Figures and Tables

**Table 1:** Primer sequences used in the study.

Name	Sequence
ALP	3′→5′: ACGAGGTCACGRCCATCCT 5′→3′: CCGAGTGGTGGTCACGAT
RUNX2	3′→5′: CCACAGAGCTATTAAAGTGACAGTG 5′→3′: ACAAACTAGGTTTAGAGTCATCAAGC
COL1A1	3′→5′: GCATGGCCAAGAAGACATCC 5′→3′: CCTCGGGTTTCCACGTCTC
CYP1A1	3′→5′: GGCCACTTTGACCCTTACAA 5′→3′: CAGGTAACGGAGGACAGGAA TCACGAT
GAPDH	3′→5′: TGGGAAGCTGGTCATCAAC 5′→3′: GCATCACCCCATTTGATGTT
